# Development of an In Vitro System to Study the Interactions of Aerosolized Drugs with Pulmonary Mucus

**DOI:** 10.3390/pharmaceutics12020145

**Published:** 2020-02-11

**Authors:** Safar Alqahtani, Clive J. Roberts, Snjezana Stolnik, Cynthia Bosquillon

**Affiliations:** School of Pharmacy, University of Nottingham, Nottingham NG7 2RD, UK; safar.alqahtani@psau.edu.sa (S.A.); clive.roberts@nottingham.ac.uk (C.J.R.); snow.stolnik@nottingham.ac.uk (S.S.)

**Keywords:** pulmonary delivery, aerosols, mucus, absorption, in vitro models

## Abstract

Mucus is the first biological component inhaled drugs encounter on their journey towards their pharmacological target in the upper airways. Yet, how mucus may influence drug disposition and efficacy in the lungs has been essentially overlooked. In this study, a simple in vitro system was developed to investigate the factors promoting drug interactions with airway mucus in physiologically relevant conditions. Thin layers of porcine tracheal mucus were prepared in Transwell^®^ inserts and initially, the diffusion of various fluorescent dyes across those layers was monitored over time. A deposition system featuring a MicroSprayer^®^ aerosolizer was optimized to reproducibly deliver liquid aerosols to multiple air-facing layers and then exploited to compare the impact of airway mucus on the transport of inhaled bronchodilators. Both the dyes and drugs tested were distinctly hindered by mucus with high logP compounds being the most affected. The diffusion rate of the bronchodilators across the layers was in the order: ipratropium ≈ glycopyronnium > formoterol > salbutamol > indacaterol, suggesting hydrophobicity plays an important role in their binding to mucus but is not the unique parameter involved. Testing of larger series of compounds would nevertheless be necessary to better understand the interactions of inhaled drugs with airway mucus.

## 1. Introduction

Development efforts in pulmonary drug delivery have primarily focused on advancing device technologies and formulations in order to improve aerosol deposition in the respiratory system as well as patients’ compliance [1]. Despite progresses in this field, no superior treatment of respiratory diseases by the inhaled route has been achieved in recent years and the attrition rate of inhaled drug candidates remains extremely high as compared to compounds developed for other therapeutic areas [2]. One of the principal underlying causes is a lack of understanding of the factors affecting drug disposition post-deposition in the lungs. Notably, very little is currently known on the interactions of inhaled drugs with the respiratory mucosa.

In the upper airways, the epithelium is covered by a ~10 μm thick mucus layer that aerosolized drugs must be able to traverse for exerting their pharmacological action as well as avoiding rapid clearance by the mucocilliary escalator. Mucus has been demonstrated to act as a permeability barrier for a range of drugs, particularly lipophilic ones, and is thus deemed to affect their efficacy [3,4]. Due to the popularity of the oral route of administration, investigations on mucus–drug interactions have nevertheless almost exclusively been performed using gastro-intestinal mucus [4] which exhibits different rheological characteristics from tracheal mucus [5], likely because of variations in their constitutive mucins and matrix organization [4]. Furthermore, study protocols have typically involved 400 μm to several mm thick mucus layers exposed to relatively large volumes of drug preparations to reproduce conditions in the gastro-intestinal tract [6,7]. Such experimental designs are not pertinent to aerosolized drugs which, in the clinic, directly land onto the airway mucosa.

Considering the mucus layer is the first biological component encountered by inhaled drugs in the upper airways, it is surprising that very few studies so far have evaluated the impact of airway mucus on drug absorption in the lungs. In the 1990s, the permeability of the model antibiotics p-aminosalicylic acid, isoniazid, and pyrazinamide was compared through a buffer solution, cystic fibrosis mucus, and pig gastric mucus [8]. Mucus secretions retarded the diffusion of all three drugs with the disease model having the greatest impact due to its higher viscosity. However, in that early work, the donor compartment of the diffusion system contained a non-physiologic large volume of drug solution. Two decades later, the permeation of gentamicin sulfate [9] and ketoprofen lysinate [10] formulated as inhalation dry powders was reported to be significantly delayed in modified Franz cells when these incorporated a layer of artificial cystic fibrosis mucus or cystic fibrosis sputum, respectively. A common limitation of the three aforementioned studies is that the thickness of the mucus layers (~3 mm) was not clinically representative. Recently, our group developed a more physiologically realistic in vitro model featuring ~100 μm thick layers of porcine tracheal mucus formed at the surface of semi-permeable Transwell^®^ cell culture inserts [11]. Salbutamol sulfate or indomethacin inhalation powders were then sprayed onto the mucus layers using a custom-made deposition system. Interestingly, mucus hindered salbutamol transport but enhanced that of indomethacin, suggesting it promoted the dissolution of poorly water soluble drugs such as indomethacin.

Herein, we intended to apply drugs onto mucus layers as solution aerosols in order to differentiate the effect of airway mucus on drug diffusion from its role on particle solubilisation. Several sophisticated devices such as the CULTEX^®^ [12], VITROCELL^®^ [13], or ALICE-CLOUD [14,15] systems have been employed to directly expose cell culture models of the airway epithelium to aerosolized droplets. However, they necessitate technical expertise and generate low-dose prolonged exposures that are more suited to environmental toxicology studies than to drug transport measurements. In contrast, the MicroSprayer^®^ IA-1C aerosolizer that was initially developed to facilitate the intratracheal administration of nebulized drugs in laboratory animals is straightforward to operate, only requires small volumes of drug formulations and delivers bolus doses alike therapeutic aerosols. Accordingly, it has successfully been used to expose cell cultures to aerosolized liquids [13,16,17,18]. The spray generated by the MicroSprayer^®^ has typically been directed towards one single cell layer. Lengthy experiments were then required to obtain a satisfactory number of replicates. Furthermore, in such configuration, cells might be damaged by the power of the spray and exposed to doses that are much higher than in a clinical scenario.

The overarching aim of this study was to develop a simple in vitro system to investigate the parameters affecting compound interactions with airway mucus in physiologically relevant conditions. We first evaluated the retardation effect of layers of porcine tracheal mucus mounted on Transwell^®^ inserts on the permeation of a range of fluorescent dyes with various physico-chemical properties. We then optimized a deposition system based on the MicroSprayer^®^ aerosolizer to simultaneously and reproducibly expose multiple biological layers to solution aerosols and applied this to determine the impact of airway mucus on the diffusion of inhaled bronchodilators. The compounds tested exhibited various permeation profiles across the mucus layers with hydrophobicity appearing as the predominant but not the sole factor driving their affinity towards airway secretions.

## 2. Materials and Methods

### 2.1. Materials

Transwell^®^ inserts (1.2 mm diameter) and well plates were purchased from Corning Ltd (Loughborough, UK). Indacaterol maleate was purchased from MedChemExpress (MCE^®^, Monmouth Junction, NJ, USA). Formic acid and HPLC grade solvents for sample analysis were purchased from Fisher Scientific (Loughborough, UK). HPLC grade water was produced with a Purelab Ultra ELGA water purification system (Veolia Water Solutions and Technologies, Birmingham, UK). Unless specified, all other chemicals and reagents were obtained from Sigma-Aldrich (Poole, UK).

### 2.2. Coating of Transwell^®^ Inserts with Porcine Tracheal Mucus

Mucus was collected from the trachea of healthy adult pigs slaughtered at a local abattoir, cleared of blood contaminants and stored at −20 °C as previously described [11]. Freezing has been demonstrated to affect neither the viscoelastic properties of native mucus nor drug coefficient of diffusion across mucus models [19]. After thawing, 12 µL of mucus were resuspended in 0.1 M NaCl to reach a final volume of 300 µL. Twelve microliters were previously found to be the lowest volume of mucus providing full coverage of the Transwell^®^ membrane [11]. The mucus suspension obtained was pipetted into Transwell^®^ inserts housed in 12-well cell culture plates. The plates were centrifuged at 1500 rpm for 15 min; the supernatant were removed and 500 µL of Hank’s Balanced Salt Solution (HBSS) were placed into the wells. The plates were stored overnight to allow the mucus to stabilize and excess water to evaporate.

### 2.3. Permeation of Fluorescent Dyes across Mucus Layers

Mucus layers were formed at the surface of 0.4 μm pore size polyester Transwell^®^ membranes as described above. Solutions (100 μM) of the fluorescent dyes Lucifer yellow CH dipotassium, Rhodamine B, Rhodamine 123, and Rose Bengal were prepared in HBSS while fluorescein isothiocyanate (FITC, 10 µM) was dissolved in HBSS containing 0.1% dimethyl sulfoxide (DMSO). Fifty μL of each dye solution pre-warmed to 37 °C were added to the apical chamber of bare inserts or inserts supporting a mucus layer and 500 μL of fresh pre-warmed HBSS (or HBSS + 0.1% DMSO in experiments with FITC) were added to the basolateral chamber. Samples (200 μL) were withdrawn from the basolateral side at pre-determined time points and replaced with 200 µL of fresh pre-warmed HBSS/HBSS + 0.1% DMSO. The plate containing the Transwell^®^ inserts was maintained at 37 °C on an orbital shaker (60 rpm) between sampling times. One hundred μL of each withdrawn sample were transferred to a black 96-well plate (Nunc F96, Scientific Laboratory Supplies, Nottingham, UK) for fluorescence measurements using a Tecan (SPARK 10M) plate-reader (Lucifer yellow: λ_ex_ = 427 nm and λ_em_ = 535 nm; FITC: λ_ex_ = 490 nm and λ_em_ = 540 nm; Rhodamine B: λ_ex_ = 544 nm and λ_em_ = 680 nm; Rhodamine 123: λ_ex_ = 540 nm and λ_em_ = 590 nm; Rose Bengal: λ_ex_ = 549 nm and λ_em_ = 620 nm). The dye concentration in the samples was determined from calibration curves prepared in HBSS or HBSS/0.1% DMSO (FITC). Experiments were performed across four mucus layers or empty Transwell^®^ inserts (n = 4). In addition, FITC transport was assessed using four different batches of mucus (N = 4; n = 4).

### 2.4. Optimization of the Aerosolization System

**Figure 1:**
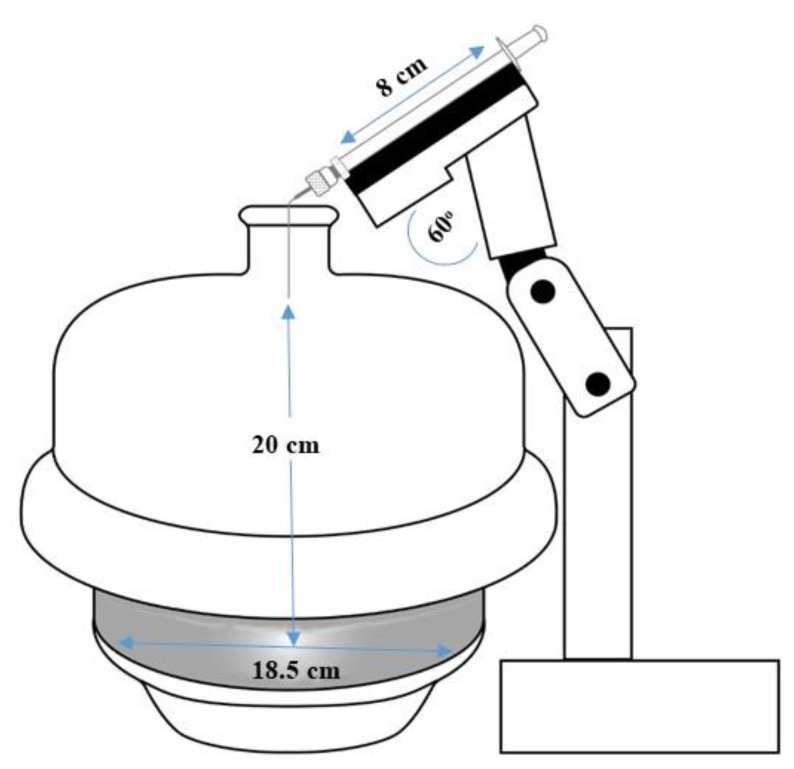
Schematic representation of the deposition system.

In order to deposit aerosolized drug solutions at the surface of the mucus layers, a MicroSprayer^®^ Aerosoliser Model IA-1C (Penn-Century. Inc. Wyndmoor, PA, USA) was mounted in a vacuum glass desiccator featuring an internal deposition surface (diameter: 18.4 cm) positioned 20.0 cm below the port entrance (Figure 1). The air-free atomizer of the MicroSprayer^®^ was secured inside a stopper and held in a vertical position facing the centre of the internal surface thanks to a custom-made external stand. Dose volume “spacers” (25 or 50 µL) were attached to the plunger of the high-pressure syringe to facilitate precise delivery of defined volumes of aerosolised solutions. To ensure the production of a uniform aerosol spray, the plunger was actuated in a sharp and firm motion.

The semi-permeable membrane of four Transwell^®^ inserts was covered with clean glass coverslips (1.2 cm in diameter, VWR, Lutterworth, UK) and those were placed inside Petri dish lids. The inserts were arranged on the internal glass desiccator surface to occupy the four corners (A, B, C, and D) of a virtual square centered below the tip of the MicroSprayer^®^ and covering a surface of 20, 50, or 100 cm^2^. A solution of Lucifer yellow (1 mM in HBSS) was loaded into the Penn-Century high-pressure syringe and various volumes (50, 100, and 200 µL) were then sprayed inside the desiccator chamber. One hundred µL of HBSS were added to each Transwell^®^, 70 μL were collected back from each insert and transferred to a black 96 well plate for fluorescence reading. Experiments were performed in triplicate for each volume sprayed and geometrical arrangement (N = 3).

### 2.5. Permeation across Mucus Following Aerosol Deposition

The transport of Lucifer yellow and various bronchodilators across mucus layers spread at the surface of 0.4 or 3.0 μm pore size polyester Transwell^®^ membrane was assessed and compared to their diffusion in corresponding empty inserts.

The MicroSprayer^®^ was loaded with Lucifer yellow (1 mM), ipratropium bromide, glycopyrronium bromide, salbutamol sulphate (all three drugs at a final concentration of 10 mM) dissolved in HBSS or formoterol and indacaterol maleate solutions (1 mM) prepared in HBSS with 10% of DMSO. A volume of 200 μL was sprayed onto mucus covered or empty Transwell^®^ inserts displayed according to the 50 cm^2^ geometrical arrangement described above. The inserts were immediately transferred to a 12-well plate and each well was filled with 500 µL of HBSS ± 10% DMSO depending on the test compound. The plate was incubated at 37 °C on an orbital shaker and samples (50 μL) were withdrawn from the basolateral chambers after pre-determined time points. Each sample was replaced with an equivalent volume of HBSS (±10% DMSO).

Following the last sampling time point, the apical chamber was washed using 200 µL of HBSS (±10% DMSO) for quantification of the residual non-permeated test compound. The washed mucus layers were collected, vortexed for one minute, then centrifuged at 14,000 rpm for 5 min and the supernatants were sampled for drug content analysis. The amount of test compound detected in the wash samples was added to the cumulative amount recovered in the basolateral chamber to estimate the dose deposited onto the mucus layers or semi-permeable membranes upon aerosolisation of the test solutions.

Lucifer yellow concentrations were determined through fluorescence measurements while drug samples were analyzed by LC-MS/MS.

Experiments were performed in triplicate (Lucifer yellow) or quadruplicate (bronchodilators) using four Transwell^®^ inserts per replicate (n = 4).

### 2.6. Drug Sample Analysis

Samples obtained from the drug permeation experiments were analyzed by LC-MS/MS.

The LC system comprised an Agilent Hewlett Packard series 1100 coupled with a Micromass Quattro Ultima Pt mass spectrometer (Waters, Milford, MA, USA) equipped with an electrospray ion source operated in positive mode. An ACE3 C18 (3 μm, 150 mm × i.d. 2.1 mm) column fitted with a C18 guard cartridge was used for all analysis.

Ipratropium bromide, glycopyrronium bromide, formoterol, and indacaterol maleate were processed similarly. All samples were diluted 1:3 with cold methanol containing 5 nM of the internal standard (glycopyrronium bromide, ipratropium bromide, indacaterol maleate, and formoterol, respectively) before being stored overnight at −20 °C. The samples were vortexed for one minute and centrifuged at 5000 rpm for five minutes at 4 °C. The supernatants were diluted (1:1) with 0.1% formic acid in water, transferred to LC-MS vials and stored in a refrigerator until analysis.

On the day of analysis, a 10 μL volume was injected into the LC-MS system. Samples were run at 0.2 mL/min applying a phase gradient, where phase A consisted of MilliQ water containing 0.1% formic acid and phase B consisted of methanol containing 0.1% formic acid. The gradient started at 45% of phase B, then increased to 90% over two minutes before returning to 45% over 3.5 min and being maintained at that level until the end of the 8.5 min run time. A source temperature of 125 °C, a desolvation temperature of 350 °C and a collision energy of 28 kV (ipratropium and glycopyrronium), 18 kV (formoterol) or 30 kV (indacaterol) were applied.

Salbutamol sulphate samples were diluted 1:1 with methanol, vortexed for a minute and centrifuged at 5000 rpm for five minutes at 4 °C. The supernatant were diluted 1:1 with phase A which consisted of an aqueous solution containing 0.1% *v/v* formic acid and ammonium formate 20 mM (pH 3.8). 50 μL of the resulting solution was injected into the LC-MS/MS system for quantification. Samples were run at 0.2 mL min^−1^ isocratically using a 50:50 mixture of phase A and methanol as the mobile phase. The source temperature was set at 125 °C, the desolvation temperature was fixed at 350 °C and the collision energy applied was 20 kV.

### 2.7. Statistical Analysis

Statistical analysis was performed using GraphPad Prism 6.02. Unpaired *t*-tests (multiple comparisons) and ANOVA two-way analysis (with Tukey’s multiple comparison tests) were used to compare data between two groups or more than two groups, respectively. Differences between experimental groups were considered significant when a *p*-value lower than 0.05 was obtained.

## 3. Results and Discussion

Due to the complexity of the lung anatomy, bio-relevant in vitro respiratory models are necessary to gain a deeper understanding of the parameters influencing the disposition of inhaled drugs following their deposition onto the airway mucosa. Mucus-drug interactions are particularly difficult to investigate in vivo, which incentivizes us to develop a model of airway mucus based on thin layers of native porcine tracheal mucus coating the semi-permeable membrane of Transwell^®^ inserts [11]. Pig mucus was used as it is similar to human mucus [20] and more readily accessible. In our previous study, we showed that our pig tracheal mucus samples exhibited similar rheological properties and internal structure as those reported for human airway secretions [11].

Porcine tracheal mucus has previously been used to assess the muco-penetrative properties of drug nanocarriers designed for the inhaled route [21]. On the other hand, its effect on the permeation of small molecules has not been investigated, at the exception of our recent study on inhalation dry powders [11]. In the present work, we assessed the role of compound physico-chemical characteristics on their permeation through porcine tracheal mucus layers, adapted our previously described deposition system [11] for production of liquid aerosols and exploited this to study the interactions of inhaled bronchodilators with airway secretions.

### 3.1. Impact of Airway Mucus on the Permeation of Fluorescent Dyes

We recently reported that mucus delayed the transport of the positively charged water soluble bronchodilator salbutamol but, in contrast, facilitated that of the negatively charged poorly soluble anti-inflammatory agent indomethacin after both drugs were exposed to the mucus layers as solid particle sprays using a bespoke deposition system [11]. As earlier studies with gastro-intestinal mucus had reported stronger binding for lipophilic molecules [3], our indomethacin data were not anticipated. They were nevertheless attributed to solubilisation enhancement properties of mucus and/or charge repulsion between the drug and the negatively charged mucin fibers. It is nevertheless possible that factors promoting compound interactions with mucus are dependent on the regional origin of the secretions. Mucus from different parts of the body are indeed well known to vary in mucin structure and scaffold organization [4].

**Figure 2:**
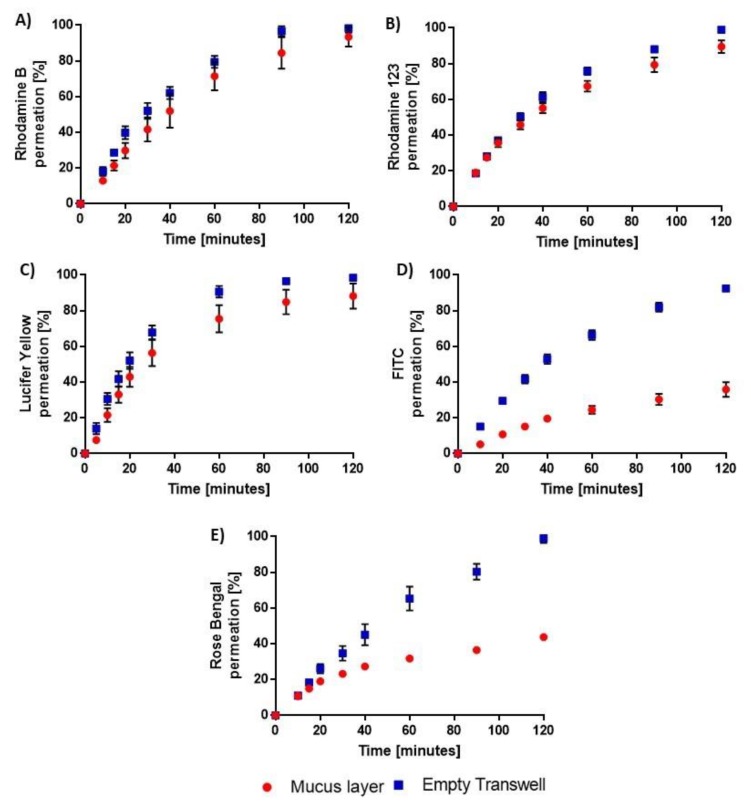
Permeation profiles of fluorescent dyes across mucus layers spread at the surface of 0.4 µm pore size polyester Transwell^®^ inserts and corresponding empty inserts. 50 µL of the dye solutions were added to the apical chambers at t = 0. **A**: Rhodamine B; **B**: Rhodamine 123; **C**: Lucifer yellow; **D**: FITC; **E**: Rose Bengal. Data are expressed as cumulative percentage of the initial donor dose recovered in the basolateral compartment as a function of time. They are presented as mean ± SD (n = 4) or mean ± SEM (FITC; N = 4, n = 4).

Therefore, we measured the diffusion of diverse fluorescent dyes encompassing a broad range of physico-chemical properties across our mucus layers or empty Transwell^®^ inserts in an attempt to uncover the molecular characteristics that might promote binding to airway mucus. Five dyes were selected for the study: Rhodamine B, a polar (logP: −1.1) amphoteric molecule; Rhodamine 123, a relatively hydrophilic (log P: 1.5) weak base that is partially ionized at pH 7.4 and three negatively charged dyes of increasing hydrophobicity; i.e., Lucifer yellow (logP: 2.6); FITC (logP: 4.5) and Rose Bengal (logP > 6). In this initial set of experiments, dye solutions were pipetted onto the layers to reflect protocols applied in related studies with gastro-intestinal mucus. Due to its poor solubility in water, DMSO was required to prepare solutions of FITC. However, the solvent did not to affect transport across the mucus layers when applied at a concentration up to 10% (Supplementary Materials, Figure S1). The reproducibility of the mucus samples was assessed by measuring FITC permeability across layers prepared from four different batches of mucus. The four separate permeation profiles of the dye were identical (Figure 2D, *p* > 0.05), demonstrating the reliability of the model.

The presence of a mucus layers at the surface of the Transwell^®^ inserts had distinct effects on the diffusion of the dyes into the basolateral chamber over time. Mucus had minimal impact on the permeation of Rhodamine B with only a slight retardation effect detected over the first 30 min of transport (Figure 2A, *p* < 0.05) and over 95% of the dose applied recovered in all receiver compartments after 120 min (Figure 2A, *p* > 0.05). The permeation profiles of Rhodamine 123 with/without mucus were identical over the first 40 min (Figure 2B, *p* > 0.05). Thereafter, small but significant differences emerged (Figure 2B, *p* < 0.05) and by the end of the experiment, 90 ± 4 (SD)% of the dye had permeated the mucus vs 99 ± 1 (SD)% for bare inserts (*p* < 0.05). In the case of Lucifer yellow, a ~10% lower diffusion across mucus was observed over its entire permeation profile (Figure 2C, *p* < 0.05), which resulted in 88 ± 7 (SD)% of the dose transported within 2 h as compared to 98 ± 2 (SD)% across empty inserts. FITC transport was more dramatically impaired by mucus as only 36 ± 4 (SEM)% of the initial dose permeated through the layers in 120 min while 92 ± 2 (SEM)% was able to cross the semi-permeable membrane of the Transwell^®^ over the same period of time (Figure 2D, *p* < 0.05 at all sampling points). Similarly, mucus markedly delayed the diffusion of Rose Bengal in the receiver chamber, reducing its permeation over 2 h from 99 ± 3 (SD)% to 43.7 ± 0.7 (SD)% of the donor dose (Figure 2E, *p* < 0.05 at all sampling points).

Despite the small number of molecules tested, hydrophobicity stood out as a key parameter driving compound interactions with airway mucus while charge had much less of an impact. This is in line with the inverse relationship previously obtained between drug diffusion coefficients in native intestinal pig mucus and their lipophilicity as well as the minor role of compound charge in mucus binding observed in that model [22]. In contrast, no correlation between transport rate and log D was found in purified pig gastric mucin [22]. It was later demonstrated that hydrophobic interactions occur preferentially with the lipids contained in raw intestinal pig mucus samples rather than with mucin itself [23].

The high concentration of lipids found in intestinal mucus, e.g., 37% in dry weight [23], is often attributed to digested food. Nevertheless, human airway mucus has also been shown to include a high lipidic fraction, i.e., 25–40% in dry weight depending on the sample [24]. Lipids were very likely present in our mucus model since only a cleaning step to remove blood contaminants was carried out during the preparation of the Transwell^®^-supported layers. Binding to lipids was therefore the most probable mechanism underlying the marked effect of pig tracheal mucus on the permeability of FITC and Rose Bengal, the two highly hydrophobic dyes tested in this study (Figure 2).

Our data suggests that low molecular weight compounds might interact with airway or gastro-intestinal mucus in a similar fashion. It would however be interesting to compare the permeation of the same series of molecules across both tracheal and gastro-intestinal mucus in identical diffusion systems. This would also indicate whether airway mucus offers any benefit in inhaled drug permeation studies or alternatively, could be substituted by gastro-intestinal mucus whose yield per animal is much higher than that of tracheal secretions.

### 3.2. Optimization of the Aerosolization System

**Figure 3:**
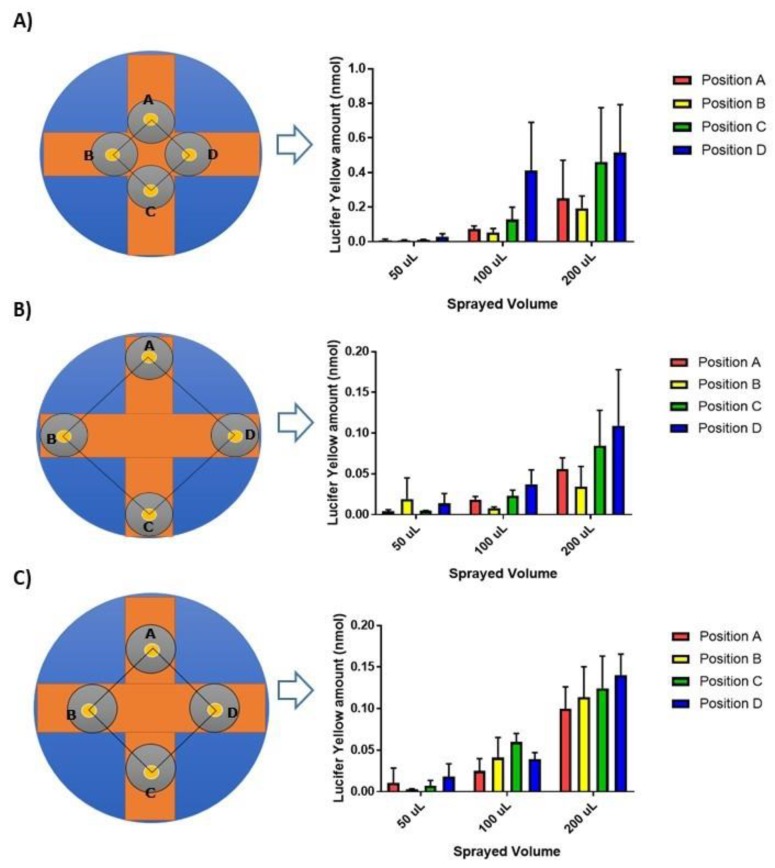
Doses of Lucifer yellow deposited on Transwell^®^ inserts when sprayed from a distance of 20 cm. Inserts were arranged to cover a 20 (**A**), 100 (**B**), or 50 (**C**) cm^2^ surface area. Data are presented as mean ± SD (n = 3).

In order to mimic the clinical situation, aerosolized drugs must be directly deposited onto any in vitro model of the airway mucosa. To achieve this, our previously described deposition system was modified by replacing the PennCentury^TM^ Dry Powder Insufflator [11] with a MicroSprayer^®^ Aerosoliser to allow the exposure of air-facing mucus layers to liquid aerosols. In order to minimize the physical impact of the liquid sprays on the mucus model, four Transwell^®^ inserts were positioned 20 cm below the tip of the Aerosoliser and at variable distance from the centre of the spray in an arrangement shaping a squared area of either 20, 50, or 100 cm^2^ (Figure 3). The extent and reproducibility of the dose delivered onto the inserts was optimized by spraying different volumes of Lucifer yellow solutions inside the aerosolization chamber.

As expected, for all geometric configurations, a spray volume of 200 µL resulted in a higher average amount of fluorescent dye delivered to the inserts than a spray of 50 or 100 µL (Figure 3). When the inserts were placed at the shortest distance from the center of the deposition platform, they received highly variable doses of the dye with any of the three volumes of Lucifer yellow aerosolized (coefficient of variation up to 72%) (Figure 3A). Positioning the Transwell^®^ inserts at the outskirts of the deposition chamber improved the reproducibly of the deposited dose and no significant difference was noted between the amount of dye reaching the four inserts when 100 µL of the solution was released from the MicroSprayer^®^ (*p* > 0.05, Figure 3B). However, that amount was very low (<0.06 nmol) and could have led to analytical challenges during permeation studies across the mucus layers. The intermediate geometric arrangement in which the inserts delineated a 50 cm^2^ square provided a compromise between efficient deposition and dose reproducibility. In that set-up, spraying a volume of 200 µL resulted in 0.12 ± 0.04 nmol (which corresponded to ~120 nL) of the fluorescent dye consistently landing onto the four Transwell^®^ inserts (*p* < 0.05, Figure 3C).

**Figure 4:**
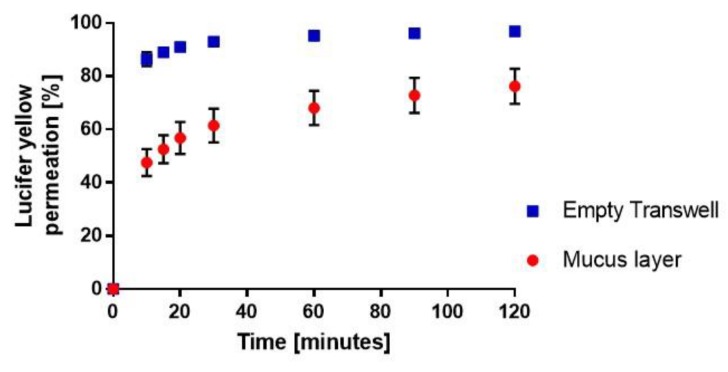
Permeation profiles of Lucifer yellow after it was sprayed at a distance of 20 cm onto mucus layers prepared in 0.4 µm pore size polyester Transwell^®^ inserts or corresponding empty inserts arranged to cover a 50 cm^2^ surface area. Data are expressed as cumulative percentage of the deposited dose recovered in the basolateral compartment as a function of time. They are presented as mean ± SEM (N = 3, n = 4).

Applying those experimental conditions, Lucifer yellow was then sprayed onto mucus layers spread at the surface of the Transwell^®^ inserts or into bare inserts and its diffusion into the basolateral compartment was monitored over 120 min. The transport of the aerosolized dye across both mucus and the semi-permeable membrane alone was initially faster than after addition of 50 μL of the dye solution into the donor chambers (Figure 2D). Indeed, after 10 min, 48% ± 5% of the deposited aerosol dose had diffused through air-interfaced mucus layers (Figure 4) as compared to 22% ± 4% (*p* < 0.05) when these were submerged by the dye solution (Figure 2D). Permeation across the bare Transwell^®^ membrane was rapid with >90% of the dose recovered in the receiver chamber after 20 min (Figure 4). This can probably be explained by the absence of a bulk solution and unstirred water layer acting as diffusion barriers upon direct exposure of the mucus or the semi-permeable membrane to the dye. However, the hindering effect of mucus on Lucifer yellow diffusion was more pronounced upon aerosolization of the dye, as indicated by the clear difference in its permeation profiles in presence or absence of airway secretions (Figure 4), unlike those across submerged mucus layers and Transwell^®^ membranes (Figure 2D). In submerged conditions, mucus could have been diluted by the dye solution and would therefore have formed a rather lose layer at the surface of the Transwell^®^ inserts allowing a freer diffusion of the solutes. This illustrates the importance of investigating drug interactions with biological barriers using in vitro systems that closely resemble the in vivo environment being modelled.

### 3.3. Interactions of Inhaled Bronchodilators with Airway Mucus

The deposition system was then employed to evaluate the influence of airway mucus on the pulmonary absorption of marketed inhaled bronchodilators of the M3 antagonist (ipratropium and glycopyrronium) and β_2_-agonist classes (salbutamol, formoterol and indacaterol). Those drugs are either cationic or zwitterionic at neutral pH but significantly differ in their lipophilicity (Table 1).

The amounts deposited onto the mucus layers ranged from ~200 to 700 ng and was more dependent on the drug itself than on the concentration of the solution loaded into the MicroSprayer^®^ (Table 1). Those variations might be related to changes in the viscosity of the test solutions and the geometry of the spray with the investigated compounds. In a clinical scenario, if one considers 20–50% of the dose emitted from the inhaler reach the lungs [25], a typical patchy drug distribution [25] and the surface area of the upper airways, the deposited dose per surface area is likely to be even lower. However, replicating such doses in vitro is not feasible as they would result in undetectable concentrations of permeated drugs.

**Figure 5:**
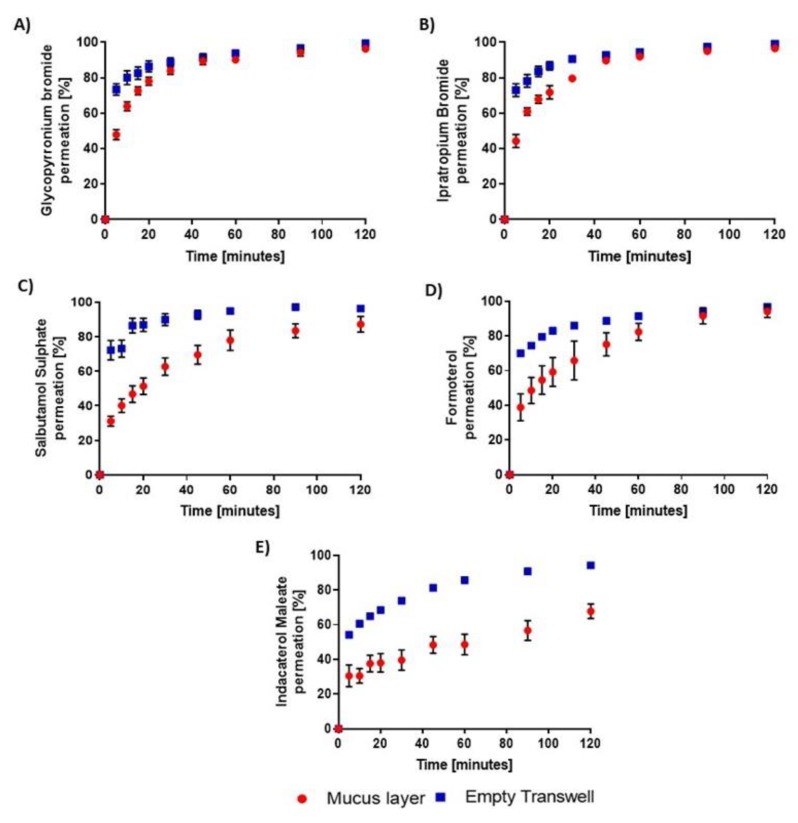
Permeation profiles of inhaled bronchodilators after they were sprayed at a distance of 20 cm onto mucus layers mounted onto 0.4 µm pore size polyester Transwell^®^ inserts or corresponding empty inserts arranged to cover a 50 cm^2^ surface area. **A**: glycopyrronium bromide; **B**: ipratropium bromide; **C**: salbutamol sulphate; **D**: formoterol; **E**: indacaterol maleate. Data are expressed as cumulative percentage of the deposited dose recovered in the basolateral compartment as a function of time. They are presented as mean ± SEM (N = 4, n = 4).

Ipratropium and glycopyrronium exhibited indistinguishable permeation profiles across the mucus layers with, respectively, 44% ± 4% and 48% ± 3% of the applied dose detected in the receiver chambers after the first five minutes and a plateau above 95% observed from 45 min onwards (Figure 5A,B, *p* > 0.05). The permeation profile of both drugs then coincided with that across the empty Transwell^®^ inserts (Figure 5A,B, *p* > 0.05). Those data were not surprising since the two M3 antagonists share similar physicochemical properties such as a low logP value and the presence of a quaternary ammonium in their chemical structure (Table 1).

In contrast, the β_2_-agonists interacted differently with airway mucus. The diffusion of the short acting salbutamol was significantly lower across airway mucus than Transwell^®^ inserts over all time points (Figure 5C, *p* < 0.05) and only 31 ± 3% of the initial dose had permeated the mucus layers within the first five minutes, confirming our previous observations that mucus impairs the pulmonary absorption of that inhaled drug [11]. The transport across airway secretions of the long-acting formoterol was faster than that of salbutamol. A higher percentage of the former drug, i.e., 39 ± 8% was recovered basolaterally five minutes post aerosolization and during the second hour of the study, the fractions that had diffused through the mucus or the bare inserts were not statistically different (Figure 5D, *p* > 0.05). Finally, a slow permeation across mucus was evident for the ultra-long-acting indacaterol (Figure 5E). While a similar amount of the drug as for salbutamol had crossed the layers within the first 5 min; i.e., 31 ± 6%, only two thirds of the applied dose of indacaterol was able to overcome the mucus barrier in 120 min (Figure 5E).

In line with previous observations with the different fluorescent dyes, the highly hydrophobic bronchodilator indacaterol was strongly retained within the mucus layers despite its net neutral charge while the highly hydrophilic positively charged M3 antagonists showed the weakest interactions with mucus. Hydrophobicity can nevertheless not be accounted for the relatively slow permeation of salbutamol across airway mucus, suggesting additional drug properties promote mucus binding, such as for instance, their ability to form hydrogen bonds with mucus components. Salbutamol indeed features a very high number of hydrogen bond acceptor and donor sites as compared to the other inhaled drugs included in this study (Table 1), which could explain its interactions with mucus were stronger than expected based on its hydrophilicity.

**Figure 6:**
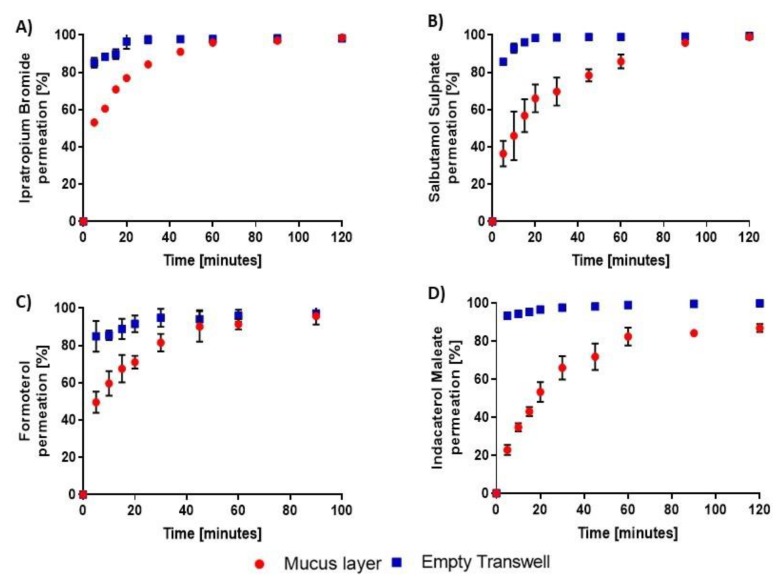
Permeation profiles of inhaled bronchodilators after they were sprayed at a distance of 20 cm onto mucus layers mounted onto 3.0 µm pore size polyester Transwell^®^ inserts or corresponding empty inserts geometrically arranged to cover a 50 cm^2^ surface area. **A**: ipratropium bromide; **B**: salbutamol sulphate; **C**: formoterol; **D**: indacaterol maleate. Data are expressed as cumulative percentage of the deposited dose recovered in the basolateral compartment as a function of time. They are presented as mean ± SEM (N = 4, n = 4).

However, as a matter of concern, it was noticeable that the Transwell^®^ membrane itself acted as a diffusion barrier to all five drugs but particularly to indacaterol (Figure 5). A similar rate-limiting diffusion across Transwell^®^ inserts had previously been reported for the poorly water soluble inhaled corticosteroid ciclesonide as part of a drug dissolution study [26]. This was then corrected by replacing the 0.4 μm pore size polyester membranes of the Transwell^®^ by glass microfiber or paper filters affixed onto the plastic walls of the inserts using mild heat [26]. As a more convenient alternative, herein, we replicated ipratropium, salbutamol, formoterol and indacaterol permeation studies with mucus layers mounted onto 3.0 μm instead of 0.4 μm pore size polyester Transwell^®^. Using those more porous inserts, at least 85% of the dose deposited onto the membrane was detected in the receiver chambers within the first 5 min for all four drugs tested (Figure 6).

Transport across airway secretions was also less restricted than when the layers were supported by semi-permeable membranes with narrower pores, as reflected by a reduction in the time needed for 50% of the applied dose to diffuse into the basolateral compartments (T_50_, Table 1). The extent of increase in transport rate was however drug dependent. The difference in T_50_ was negligible for ipratropium but pronounced for indacaterol (Table 1), suggesting that molecular diffusion through the Transwell^®^ membrane can be delayed due to hydrophobic interactions. Nevertheless, the four bronchodilators ranked in the same order in terms of diffusion rate across mucus in the two types of inserts; i.e., ipratropium > formoterol > salbutamol > indacaterol (Table 1), which indicates that mucus had overall a more significant impact on their permeation than the semi-permeable membrane. It is noteworthy that, in contrast to the pore size, the membrane material had a very limited influence on drug transport. Indeed, the permeation profiles of indacaterol with or without mucus in polycarbonate vs polyester Transwell^®^ were comparable, although the drug was marginally more hindered in the former (Supplementary Materials, Figure S2). This is not surprising considering the two polymers exhibit close water contact angles; i.e., 84° for polycarbonate [27] and ~75° for polyethylene terephthalate (PET, polyester) [28]. Accordingly, they are both considered as moderately hydrophilic with polycarbonate being slightly more hydrophobic.

Overall, our data suggest that airway mucus could interfere with the absorption of inhaled bronchodilators in the lungs. The impact of mucus on drug diffusion may nevertheless be overestimated in this model. Indeed, the thickness of the mucus layers (~100 µm) was 10 fold that of the mucus blanket in vivo as it was not experimentally possible to form thinner supported mucus layers that covered the entire surface of the Transwell^®^ membranes [11]. Furthermore, inhaled bronchodilators all show a very low permeability across bronchial epithelial cells in vitro [11,29,30,31] and we previously reported salbutamol transport was restricted to a larger extent by epithelial layers than by mucus [11]. Therefore, the airway epithelium likely represents the major barrier to absorption for those drugs in the lungs. Binding to the mucus layer could nevertheless enhance the retention of relatively hydrophobic inhaled drugs in the pulmonary tissue, although this would presumably be associated with a loss of therapeutic activity.

## 4. Conclusions

A simple deposition system was assembled to deliver reproducible doses of liquid aerosols to multiple Transwell^®^ inserts. It was used in this work to explore the impact of airway mucus on the permeation of a series of inhaled bronchodilators in physiologically relevant conditions but would also be suitable for exposing air-interfaced respiratory cell culture models to aerosolized drugs and formulations. Airway mucus was shown to delay the diffusion of all tested drugs, although to various extents. The number of compounds included in the study was too small to establish reliable correlations between physico-chemical properties and mucus affinity. Nevertheless, hydrophobicity emerged as one important factor. The involvement of additional drug characteristics was evident although defining these will require measuring the transport of larger series of compounds across airway mucus. This study indicates binding to mucus might affect the disposition and therapeutic activity of inhaled drugs in the lungs. Investigating their interactions with airway mucus is therefore worth pursuing, ideally using mucus from diseased rather than healthy lungs.

## Figures and Tables

**Table 1:** Properties, dose deposited and permeation rate through mucus layers of the bronchodilators tested.

Compound	Chemical Class	LogP *	MW *	H-Bond (Donor/Acceptor) *	Dose Deposited (ng)	T50 ^a^ (min)	T50 ^b^ (min)
Ipratropium	Quaternary	−1.8	412	1/4	442 ± 76	7.0 ± 0.5	<5
Glycopyronnium	Quaternary	−1.4	398	1/4	384 ± 92	5.4 ± 0.9	ND
Salbutamol	Base	1.4	577	10/12	687 ± 116	19 ± 3	12.4 ± 0.8
Formoterol	Base	2.2	344	4/5	216 ± 46	13 ± 3	7 ± 1
Indacaterol	Zwitterion	4.05	509	6/8	232 ± 61	67 ± 11	20 ± 1

* extracted from PubChem (https://pubchem.ncbi.nlm.nih.gov); MW: molecular weight; T_50_: time taken for 50% of the deposited dose to cross mucus layers mounted on ^a^ 0.4 μm or ^b^ 3.0 μm pore size inserts; ND: not determined; Data are presented as mean ± SEM (N = 4, n = 4).

## Supplementary Materials

The following are available online at https://www.mdpi.com/1999-4923/12/2/145/s1, Figure S1: Effect of increasing concentrations of DMSO on solute transport across mucus layers, Figure S2: Effect of the Transwell^®^ membrane composition on molecular diffusion.
