# Supplementary material for: Development of an In Vitro System to Study the Interactions of Aerosolized Drugs with Pulmonary Mucus
Source: Pharmaceutics. 2020 Feb 11;12(2):145. doi: 10.3390/pharmaceutics12020145 (PMC7076363; doi:10.3390/pharmaceutics12020145)
Supplement: Supplementary file 1 [file pharmaceutics-12-00145-s001.pdf]

Supplementary info

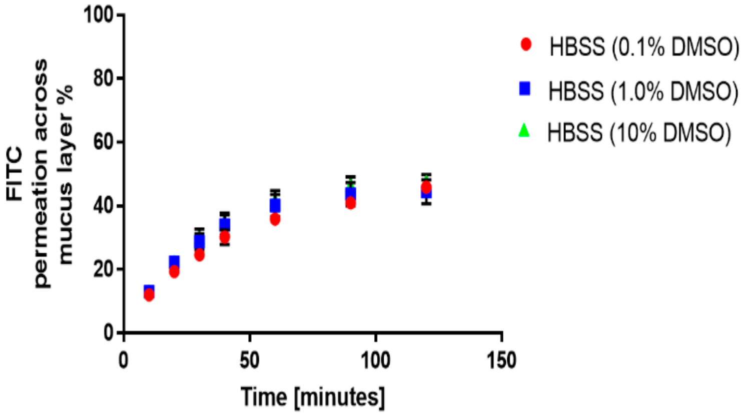


**Figure S1.** Permeation profile of FITC across mucus layers mounted onto 0.4 µm pore size polyester Transwell® inserts. FITC was dissolved in HBSS containing increasing concentrations of DMSO and added to the apical compartment as a 50 µL solution. 500 µL of HBSS containing the same DMSO concentration as the donor solution was present in the basal compartment. Data are expressed as cumulative percentage of the donor dose recovered in the basolateral compartment as a function of time. They are presented as mean ± SD (n = 4).

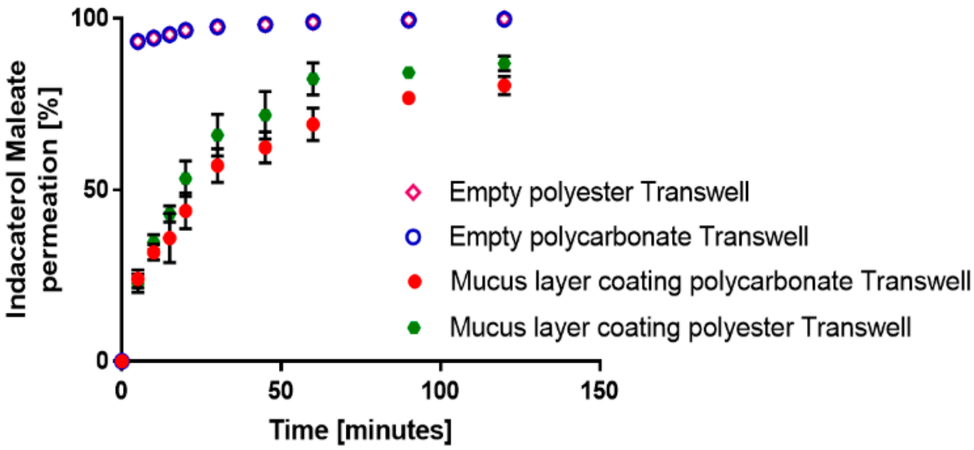


**Figure S2.** Permeation profile of indacaterol maleate across mucus layers mounted on polyester or polycarbonate 3.0 µm pore size Transwell® inserts and corresponding empty inserts. Indacaterol maleate was sprayed at a distance of 20 cm onto inserts geometrically arranged to cover a 50 $cm^2$ surface area. Data are expressed as cumulative percentage of the deposited dose recovered in the basolateral compartment as a function of time. They are presented as mean ± SEM (N = 4, n = 4).
